# Stress, stressors and related factors in clinical learning of midwifery students in Iran: a cross sectional study

**DOI:** 10.1186/s12909-020-1970-7

**Published:** 2020-03-18

**Authors:** Behrooz Rezaei, Juliana Falahati, Raziyeh Beheshtizadeh

**Affiliations:** 1 Nursing Department, Nursing & Midwifery Faculty, Falavarjan Branch, Islamic Azad University, Isfahan, Iran; 2 Midwifery Department, Nursing & Midwifery Faculty, Falavarjan Branch, Islamic Azad University, Isfahan, Iran; 3 Isfahan Health Center N. One, Isfahan University of Medical Sciences, Isfahan, Iran

**Keywords:** Clinical learning environment, Clinical education, Clinical Preceptorship, Midwifery education, Stress, Student, Midwifery, Instructor

## Abstract

**Background:**

Midwifery is an emotionally challenging profession, and academic education of midwifery especially clinical learning has its own specific challenges. Midwifery students face with stressful experiences, especially related to instructor and characteristics of clinical environment, which can affect their theoretical and practical abilities. There is insufficient evidence in this field. This study aimed to explore (1) the perceived stress and stressors of midwifery students and (2) the relationships between students’ stress and related factors in clinical learning environment.

**Methods:**

A cross sectional, survey design was conducted at one university in Iran. A sample of 108 students was selected using Krejcie and Morgan table in 2016. Data was collected using Persian version of Cohen’s perceived stress scale, Persian questionnaire of sources of stress and demographic form. Data was analyzed using independent t, ANOVA and correlation coefficient test (α < 0.05).

**Results:**

Participants returned 70 surveys (response rate, 64.8%). Approximately 56% of the students perceived a high level of stress. The most common dimensions of stressors were “unpleasant emotions” and “humiliating experiences”. The highest stressors were included “feeling suffering due to seeing for patients with critical situation”, “instructor’s admonition in the presence of clinical staff” and “communication with instructor”. The “interest in the field of study” had a negative impact on perception of stressors in dimensions of “clinical practices” and “interpersonal communication”.

**Conclusions:**

The midwifery students reported their stress in severe level, especially in dimensions of “unpleasant emotions” and “humiliating experiences”. The factors associated with the instructors have caused more stress in students. These findings will highlight need for supportive strategies by the clinical instructors. In this regard, the use of experienced instructors, the development of communication skills of the instructors, increasing coping skills of the students and the creation of a supportive environment may be helpful.

## Background

Stress is a force and a stressor is something that applies stress to an individual. Many studies have shown that stressors have a negative effect on individual’s performance and health. There are at least three distinct stressors in the field of education; the learning expected, learning environment, and the instructors [1]. Learning and practice in health sciences including midwifery are intrinsically stressful [1–4]. Midwifery is an emotionally challenging profession, and midwifery education has its own specific challenges [5–7]. Stress is a pervasive problem in midwifery education which affects the theoretical and practical learning [2–4]. This stress stems from theoretical and clinical education, but it is mainly related to clinical learning [4, 6, 8]. Although midwives say midwifery is inherently stressful, mostly contextual and environmental factors cause this stress [9]. In such a challenging environment, midwifery students experience high stress due to organizational and educational sources [10].

The maternity ward is a challenging environment that plays an important role in developing the competence of midwifery students [5, 8]. Clinical learning environment is an integral part of midwifery education program, accounting for half of the curriculum [6, 11]. The midwifery instructors play a major role in improving the students’ competency and creating their professional skills through making learning opportunities and positive experiences [4–6, 12]. Any obstacle in clinical environment can interfere with the effectiveness of clinical learning and lead to undesirable experiences for students [6, 11]. The presence of a large number of students in the clinical setting, the lack of coordination between clinical staff and instructor, the lack of experienced instructors, fear of doing harm and encountering the first childbirth, fear of penalty, lack of adequate training facilities and the lack of suitable support of students by instructors and midwives are the most causes of educational stress of Iranian midwifery students [5, 6, 8, 13]. These stresses impact on academic performance and lead to physical and psychological disorders [14, 15] and impede adaptation with the professional role [8]. Among all the factors, instructors have a crucial role in clinical learning and providing enough support for the students in clinical settings [2, 16].

As far as we know, a plethora of studies have been conducted in nursing education but the studies in midwifery education are insufficient [2, 4, 6, 12, 16–21]. A few of national studies have investigated midwifery students’ perspective on the perceived feed-back in clinical education [22], experiences of learning clinical skills [13] and experience of fear in the clinical environment [8]. Although there is a strong focus on interventions to reduce stress of midwifery students, the research show that midwifery students still experience significant stress and anxiety in clinical settings [2, 4, 6, 8, 12, 13, 16–21]. Improving the quality of clinical learning requires the provision of an appropriate learning environment. One of the prerequisites to this environment is identifying the educational stressors through feedback from students [4, 6]. Therefore, more student-centered research is needed to identify stressors, barriers, and facilitators of educational stress [2]. There is not sufficient evidence to define the state of midwifery student mental health especially stressors in clinical education, when compared to other students or healthcare professionals [23]. There is a growing need for improving the clinical learning environment. By identifying sources of stress, strategies for adapting students to stressful clinical situations can be designed and appropriate opportunity for learning can be provided. Therefore the aims of this study are:

- To explore perceived stress and its relationship with some individual and educational characteristics of the midwifery students in the clinical learning environment.
- To explore sources of stress and their relationships with some individual and educational characteristics of the midwifery students in the clinical learning environment.

## Methods

### Ethical statement

Written informed consent to enter the study, anonymously and, confidentiality of the information, and the right to withdraw from the study were the ethical considerations in this study. The questionnaires were written and anonymous, and the researchers first stated the goals of the study to the students, then obtained written informed consent from the participants, and eventually distributed the questionnaires to the participants.

### Study design

A cross-sectional survey design was performed, containing a structured questionnaire, which was completed during May 2016. A cross-sectional survey collects data to make inferences about a population of interest at one point in time.

### Setting and context

In Iran, the birth rate has increased from 2001 to 2015, but has declined since 2015. According to the national census in 2015, Iran’s population growth rate was 1.24%, with more than 1500,000 births in this year. The majority of deliveries are performed in the hospital by a gynecologist or a female midwife. The provision of maternity care in labor units is mostly organized by the medical model, and the midwives practice under the supervision of obstetricians. There are few cases of the midwifery-led model in which autonomous midwives attend the births of their clients [13]. Therefore midwives have little opportunity to gain experience in providing midwifery care in hospitals independently. Midwifery career status shows that midwifery students have very little chance of acquiring independent experience in practical activities. This leads to unpleasant fears and experiences and increases stress during clinical education [8, 13]. Past studies have shown that midwifery students were not satisfied with clinical skills, supervision, and access to information prior to clinical education [8, 24].

The undergraduate midwifery course in Iran is a four-year university program. The admission process for studying undergraduate midwifery is based on a competitive national examination. Almost all of the midwifery students are female and school leavers. They have not enough information about hospital environments and the future of midwifery profession before entering their course [13]. From the second semester, their clinical learning is begun by supervising the clinical instructors in the clinical settings [13]. The common method of clinical education for midwifery students in Iran is teacher-centered. This method may lead to inadequate clinical skills [24]. The more of clinical instructors are full- time academic members that are responsible for providing students’ theoretical courses as well as clinical education. Some of them are also part-time members who have BS or MS degrees in midwifery, and who only cooperate in clinical education [8].

### Subjects and sampling

The population of the study was undergraduate midwifery students from one of the nursing & midwifery facultiy in Isfahan province, Iran (*N* = 150). A sample of 108 students was selected during May 2016 using Krejcie and Morgan table. If the volume of the community is known, the easiest way to determine the sample size is to refer to the Morgan table [25]. The inclusion criteria included passing at least one semester of internship in the clinical setting. The exclusion criteria included students who had anxiety and stress which was diagnosed by a doctor, and were taking medication according self reporting. The questionnaires which were not filled completely were also excluded from the study. Finally, only 70 of 108 students passed inclusion/exclusion criteria, and their data was used in statistics analysis.

The participants were selected using stratified random sampling [26]. In First stage, students in each educational semester were considered as a stratum (including 4 stratums). Then, the sample size in each stratum (semester) was calculated (stratum1 = 18, stratum2 = 14, stratum3 = 35, stratum4 = 21). Finally, the students were coded in each semester and samples of each semester were selected using simple random sampling.

First, permission from the hospitals’ management and the faculty management were obtained. In second, participants received information about the study and consent (e.g. the aims of the study, voluntary participation, confidentiality, anonymity, and right to withdraw the study). Then, participants who had informed consent for the study completed a paper version of unnamed questionnaires distributed by the researchers. Finally, the data of 70 participants were analyzed (response rate: 64.8%).

### Research instruments

Two scales were used to collecting data after permission; The Persian version of Cohen’s Perceived Stress Scale (PSS) and the Persian Questionnaire of Stressful Sources (PQSS) in clinical learning. In addition, demographic and educational characteristics of the students (i.e. age, marital status, place of residence, semester, grade point average, interest of students in their field of study, number of clinical units completed) and the instructors (i.e. education level, educational work experience, clinical work experience, employment type) and the clinical environment (i.e. type of the hospital and the ward in which students were being learning clinical activities) were gathered by a self reported demographic questionnaire .

PQSS was designed by Iranian researchers [20], and evaluates the sources of stress in clinical learning environment from nursing and midwifery students’ view point. This questionnaire consists of 29 items in four-point Likert scale (from “never = 1” to “always = 4”). The PQSS measure sources of stress in four dimensions including: interpersonal communication (8 items), clinical practices (8 items), unpleasant emotions (7 items) and humiliating experiences (6 items). The score of PQSS ranges from 29 to 116, and the higher score indicated the perception of higher sources of stress. Test-retest reliability and content validity of this tool have been confirmed in some national studies and the Cronbach’s alpha has been reported to be 0.81 to 0.89 [20, 21, 27]. In this study, the reliability of this tool was confirmed by a Cronbach’s alpha coefficient of 0.94.

The Persian version of the PSS designed by Cohen et al. [28] was used. This version has 14-item. This tool is employed to determine the individual’s level of perceived stress in response to unpredictable events during the past month. The items are responded to based on a 5-point Likert scale (from “never = 0” to “all time = 4”). The eight items in this questionnaire are reverse scored. The score of PSS ranges from 0 to 56. The higher score indicates higher perceived stress. The total scores of the PSS are classified into three levels of stress; low (score 0–14), moderate (score 15–28), and severe (sore 29–56). In the study by Cohen et al., the coefficient of internal consistency has been reported 0.84 to 0.86 [28]. The reliability of the Persian version of this tool has been confirmed with the alpha coefficient of 0.89 [21].

### Statistical analysis

We used descriptive statistics using means, standard deviations (SD), minimum, and maximum for continuous variables (i.e. age, number of clinical units completed, educational work experience of instructor, clinical work experience of instructor, the scores of PSS and PQSS). Frequency and percentage were used for describing categorical variables (i.e. marital status, interest in the field of study, ward and hospital type of clinical learning environment, employment and education of instructor). The Kolmogorov-Smirnov test was used to verify the normality of the data. In the initial analysis, the scores of PSS and PQSS did not passed some of the hypotheses required for linear regression models (including: homoscedasticity, uncorrelated errors, and remaining normality) [29]. So, in order to determine the association between the scores of PSS and PQSS and the continuous variables we used independent t-test, one-way analysis of variance (ANOVA) and correlation coefficient tests. All statistical tests were carried out at 95% confidence level using SPSS/21 software (SPSS Inc., Chicago, IL, USA).

## Results

The data for 70 participants were analyzed (response rate: 64.8%). According to the results 68.6% of the students were less than 22 years of age and the majority (78.6%) was single. The average of grade point average (GPA) was 16.32 ± 1.37 and the average number of clinical units completed was 10.86 ± 6.44. Also, the average of educational work experience and clinical work experience of the instructors were (9.96 ± 7.50) and (10.40 ± 7. 23) respectively (Table 1).

**Table 1:** Individual and educational characteristics of the midwifery students

Variable	Category	N (%)
Semester	4th	14 (20.0)
	5th	10 (14.3)
	6th	32 (45.7)
	7th & 8th	14 (20.0)
^a^Grade point average	< 15	12 (17.1)
	15–17	35 (50.0)
	17<	23 (32.9)
	Low	3 (4.3)
Interest of students in their field of study	Medium	29 (41.4)
	High	38 (54.3)
	Living with family	62 (88.6)
Place of residence	Living alone	3 (4.3)
	Living in dormitory	5 (7.1)
	22>	48 (68.6)
Age (years)	22–25	17 (24.3)
	25<	5 (7.1)
Marital status	Single	*55 (78.6)*
	Married	15 (21.4)
Ward type	Labor	41 (58.6)
	Women and Midwifery ward	18 (25.7)
	Midwifery clinic	11 (15.7)
Hospital type	University Hospital	15 (21.4)
	District Hospital	44 (62.9)
	Community Health centers	11 (15.7)
Education of instructor	Bachelor degree	64 (91.4)
	Master degree	6 (8.6)
Employment of instructor	Full -time	23 (32.9)
	Part-time	47 (67.1)

^a^The acceptable minimum grade point for each lesson is 10 (range 0 to 20) and the average grade point average for course is 12. In addition, after passing all theoretical and clinical courses, the student must pass the final clinical exam and acquire a minimum score of 12 (Source: Midwifery Undergraduate Curriculum, Iranian Supreme Medical Sciences Planning Council, Available at: http://mbs.behdasht.gov.ir/index.aspx?siteid=176&fkeyid=&siteid=113&pageid=18442)

The Kolmogorov-Smirnov test showed that the scores of PSS (z = 0.718, *p* = 0.682) and PQSS (z = 0.706 and *p* = 0.702) had normal distribution. The mean score of PSS was (29.81 ± 7.68), indicating a severe stress level. In detail, 55.7% of the students reported their stress at a high level, 41.4% at a moderate level and 2.9% at a low level.

In this study, PSS’ score was not significantly different based on characteristics of the students (i.e. age, semester, interest in the field of study, GPA, place of residence, marital status, and the number of clinical units completed), the instructors (type of employment, education level, educational work experience and clinical work experience) and the clinical environment (type of hospital and type of ward) (Tables 2 and 3).

**Table 2:** The scores of PSS in terms of some individual and educational characteristics of midwifery students

Variable	Category	Mean ± SD	Statistics
Age (Years)	22>	29.38 ± 8.24	*F* = 0.921
	22–25	29.77 ± 5.15	*P* = 0.403
	25<	34.20 ± 7.40	
Marital status	Single	29.67 ± 7.73	*t* = −0.298
	Married	30.33 ± 7.08	*p* = 0.767
Grade Point Average	15>	29.58 ± 7.93	*F* = 0.524
	15–17	30.69 ± 7.84	*P* = 0.595
	17<	28.61 ± 7.06	
Semester	4th	26.43 ± 8.13	*F* = 1.431
	5th	30.30 ± 5.33	*P* = 0.242
	6th	31.44 ± 8.52	
	7th & 8th	29.36 ± 5.06	
Interest of students in their field of study	Low	20.00 ± 7.21	*F* = 2.822
	Medium	29.96 ± 6.59	*P* = 0.067
	High	30.47 ± 7.91	
Place of residence	Living in dormitory	28.40 ± 10.64	*F* = 0.939
	Living with family	29.90 ± 7.74	*P* = 0.748
	Living alone	29.25 ± 4.71	
Number of clinical units completed	5>	26.29 ± 7.39	*F* = 2.617
	5–9	30.18 ± 5.20	*P* = 0.058
	10–15	32.54 ± 9.03	
	15<	27.85 ± 5.84	
	Total mean score of PSS	29.81 ± 7.56

**Table 3:** The scores of PSS in terms of other individual and educational characteristics of midwifery students

Variable	Category	Mean ± SD	Statistics
Education level of instructor	Bachelor degree	29.80 ± 7.32	*t* = − 0.030
	Master degree	29.87 ± 8.64	*p* = 0.976
Employment type of instructor	Full-time	32.33 ± 7.73	*t* = 1.469
	Part- time	29.13 ± 7.43	*p* = 0.147
Educational work experience of instructor (years)	5>	30.43 ± 7.14	*F* = 1.869
	5–15	32.40 ± 8.36	*P* = 0.162
	15<	27.30 ± 7.66	
Clinical work experience of instructor (years)	5>	29.77 ± 7.55	*F* = 1.808
	5–15	32.21 ± 7.67	*P* = 0.172
	15<	27.71 ± 7.18	
Ward type	Midwifery clinic	32.27 ± 6.90	*F* = 2.441
	Labor	28.17 ± 6.57	*P* = 0.095
	Women and Midwifery ward	32.06 ± 9.30	
Hospital type	University Hospital	29.44 ± 7.51	*F* = 0.849
	Distract Hospital	29.30 ± 7.69	*P* = 0.432
	Community Health Center	32.70 ± 7.12	
	Mean score of PSS	29.81 ± 7.56	

Considering different number of items in the dimensions of PQSS, the average score for each dimension was calculated based on a Likert scale (1–4) and then these dimensions were then compared (Table 4). The average of total scores of PQSS was 2.14 ± 0.50. The dimensions of “unpleasant feelings” (2.46 ± 0.88) and “humiliating experiences” (2.11 ± 0.74) gained the highest scores. There was a significant difference between the scores of domains of the PQSS (*p* = 0.001 *f* = 11.92).

**Table 4:** Average scores of PQSS in clinical training in four domains (range = 1–4)

Domain of PQSS (Mean ± SD)	Ranking the domains	The lowest stressor in each domain (Mean ± SD)	The highest stressors of each domain (Mean ± SD)
Interpersonal Communication		Communication with clinical staff	Communication with instructor
(1.95 ± 0.55)	4	(1.21 ± 0.48)	(2.49 ± 1.06)
			Communication with head nurse
			(2.21 ± 1.05)
Clinical practices (2.02 ± 0.66)	3	Administering oral medication (1.59 ± 0.75)	Seeing a dead neonate (2.40 ± 1.22)
			Care for a acute patient
			(2.39 ± 1.07)
Unpleasant emotions	1	Contradiction with patients and their family	Feeling suffering due to see patients with critical situation
(2.46 ± 0.68)		(1.81 ± 0.79)	(2.96 ± 0.88)
			Inadequate care from the midwife
			(2.80 ± 0.89)
Humiliating experiences	2	Preparing the patient bed	Instructor’s admonition in the presence of doctors and staff
(2.11 ± 0.74)		(1.51 ± 0.68)	(2.70 ± 1.05)
			Instructor’s admonition in the presence of students
			(2.50 ± 1.05)

In addition, the highest stressors in the dimensions of “unpleasant feelings” and “humiliating experiences” are related to “feeling suffering due seeing patients with critical situation” (2.96 ± 0.88) and “instructor’s admonishing in the presence of clinical staff and doctors” (2.70 ± 1.05) respectively. Furthermore, the highest stressor in the dimension of “interpersonal communication” was related to “communicating with the instructor” (2.49 ± 1.06) (Table 4).

Assessing the correlation between the scores of PQSS and the characteristics of the students showed that “interest to the field of study” had a negative correlations on students’ perception of stressors in dimensions of clinical practices (*p* = 0.003, *r* = − 0.350) and interpersonal communication (*p* = 0.022, *r* = − 0.153) (Table 5).

**Table 5:** Pearson and Spearman correlation coefficient tests between the students' individual and educational characteristics with the total scores of PQSS and its domains, and score of PSS

Variable	Humiliating experiences (*r* / *p*)	Unpleasant emotions (*r* / *p*)	Interpersonal Communication (*r* / *p*)	Clinical practices (*r* / *p*)	Total score of stressful resources (*r* / *p*)	The score of PSS (*r* / *p*)
Age^a^	*r* = −0.070	*r* = −0.015	*r* = −0.093	*r* = −0.203	*r* = −0.130	*r* = 0.145
	*P* = 0.563	*P* = 0.901	*P* = 0.442	*P* = 0.092	*P* = 0.283	*P* = 0.231
Interest of students in their field of study^b^	*r* = −0.044	*r* = −0.026	*r* = −0.153	*r* = −0.217	*r* = −0.128	*r* = 0.170
	*P* = 0.514	*P* = 0.692	***P*= 0.022**	***P*= 0.001**	*P* = 0.054	***P*= 0.011**
Semester^b^	*r* = −0.027	*r* = −0.096	*r* = −0.025	*r* = −0.213	*r* = −0.085	*r* = 0.121
	*P* = 0.826	*P* = 0.427	*P* = 0.840	*P* = 0.076	*P* = 0.482	*P* = 0.320
Grade Point Average^a^	*r* = 0.081	*r* = 0.072	*r* = 0.026	*r* = 0.167	*r* = 0.099	*r* = −0.173
	*P* = 0.511	*P* = 0.552	*P* = 0.832	*P* = 0.172	*P* = 0.423	*P* = 0.158
Number of clinical units completed^a^	*r* = 0.067	*r* = −0.053	*r* = 0.0020	*r* = −0.200	*r* = −0.019	*r* = 0.138
	*P* = 0.579	*P* = 0.664	*P* = 0.987	*P* = 0.098	*P* = 0.875	*P* = 0.225
Clinical work experience of instructor^a^	*r* = 0.058	*r* = 0.088	*r* = −0.037	*r* = −0.038	*r* = −0.055	*r* = −0.148
	*P* = 0.631	*P* = 0.469	*P* = 0.763	*P* = 0.755	*P* = 0.652	*P* = 0.220
Educational experience of instructor^a^	*r* = 0.099	*r* = 0.100	*r* = 0.055	*r* = 0.002	*r* = 0.016	*r* = −0.109
	*P* = 0.416	*P* = 0.410	*P* = 0.653	*P* = 0.989	*P* = 0.896	*P* = 0.371

^a^Pearson correlation coefficient test

^b^Spearman correlation coefficient test

The total scores of PQSS had not significant correlations with marital status (*t* = − 1.152, *p* = 0.253) and place of residence (*f* = 0.647, *P* = 0.588). In addition the total scores of PQSS had not significant association with education level (*t* = 0.57, *p* = 0.955) and type of employment (*t* = − 0.211, *p* = 0.833) of the instructors.

## Discussion

The results showed that more than half of the students reported their perceived stress at a severe level. The most common dimensions of educational stressors were “unpleasant emotions” and “humiliating experiences”. The highest stressors were included “feeling suffering due to seeing for patients with critical situation”, “instructor’s admonition in the presence of clinical staff” and “communication with instructor”. The “interest in the field of study” had a negative impact on perception of stressors in dimensions of “clinical practices” and “interpersonal communication”.

In considerable research the educational stress of nursing and midwifery students were reported at a moderate or severe level [14, 18, 21, 30–32]. The study of Geraghty, Speelman & Bayes showed that with the increase in the number and severity of stressors, adverse effects on midwives are increased, and their job commitment and job engagement diminish [9]. High stress levels in students may be due to multiple stressors such as poor health of clients, complexity of care, lack of knowledge and clinical skills of the students, ineffective communication between instructor and the students, supervision style of the instructor, and gap between theory and practice. The stress of students and their stressors in learning environment can vary according to the field of study, nationality and region [30].

Clinical learning is the main part of midwifery curriculum [12]. Since the clinical environment is one of the most stressful education environments [6], students experience a wide range of problems during the clinical learning [16]. Fear of doing harm, fear of facing the first labor and fear of punishment are the factors enhancing the stress of midwifery students [8]. These stresses have potentially a negative effect on students’ learning [11, 12], cause poor clinical performance, and lead to physical and psychological problems [6, 8]. Although midwives say midwifery is inherently stressful, mostly contextual and other environmental factors cause stress in midwifery [9].

Since health of midwives and midwifery students directly affects the quality of maternal care, these stresses must be adequately moderated by instructors’ support [10]. The focus of instructors on the psychological complexities of midwifery role can be effective in adjusting and normalizing the inevitable tensions and inherent problems of this profession [7]. It seems the best way forward would be education for clinical instructors in how better to support the clinical learning of future midwives.

In present study, there were no significant relationships between the perceived stress with individual and educational characteristics of the students. In some previous studies, perceived stress were not significantly associated with age [15], gender [21], and academic semester [21, 31]. However, Smith & Yang showed that students in their final year reported the highest stress [33]. Discrepancies in these findings can be due to differences in the field of study of the participants, clinical environment and characteristics of the subjects under study. However, stress is a recognized problem in the lives of nursing and midwifery students [33], which occurs mainly due to the stressful nature of educational environment in these professions. To provide a desirable clinical environment, school managers and instructors should be aware of the facilitators and barriers to improving the clinical learning environment [11]. Counseling about managing stress, colleagues’ support, the families’ support and providing a supportive clinical environment may be useful in preventing stress and its negative consequences [30]. Recognizing and enhancing coping skills in undergraduate midwifery students can help them to adapt to different stressors during their clinical learning [17, 30]. It is suggested that, before the outset of the clinical learning course, students should be informed of the potential stresses during their course, and they would be supported properly by their instructors and other clinical staff.

The results showed there was no significant relationship between perceived stress and the instructors’ characteristics were studied (including type of employment, educational level, educational work experience and clinical work experience). Some previous studies reported that other characteristics of instructors have affected on the stress levels of students. In the study by Najafi Dolatabad et al., factors related to instructors showed a stronger relationship with stress compared to the individual characteristics of students [19]. In another study the instructor’s supervision style was effective on perceived stress of the students [34]. We expected that some of the characteristics of instructors including educational and clinical work experience were related to student stress. But the results of the present study were different. Perhaps because the characteristics of the instructors examined in our study were different. It seems that in the stressful environment of clinical learning which the majority of the students have experienced a high level of stress, educational work experience and clinical work experience of the instructors had no modified the students’ stress level. Of course, this requires further research. However mutual interaction and understanding and shared goals are essential for the positive experience of students in an optimal learning environment [35]. School managers need to assess and enhance the communication skills of midwifery instructors to reduce students’ stress [19].

The students reported the most stressors in the “unpleasant feelings” and “humiliating experiences” domains. In this regard factors such as “feeling suffering due to seeing patients with critical situation”, “instructor’s admonition in the presence of staff and doctors” and “communication with instructor” were reported as the highest stressors. Similarly, Poorheidari et al. reported that the highest domain of stressors was related to the “unpleasant feelings” domain, and “instructor’s admonition in the presence of the patient” and “lack of the instructor’s support” were the highest stressors [6]. Rafati et al. indicated that one of the main areas inducing stress was ineffective communication between instructor and students [36]. Thunes et al. also reported that the most important factor affecting students’ well-being and learning is their relationships with their instructor [35]. Anyway we have to say that academic, interpersonal and environmental stressors are direct stressors among midwifery students [37] which among them, interpersonal stressors make more students’ stress than other factors.

Midwifery students experience problems during their academic and clinical courses, which leading to uncertainty, dissatisfaction and lack of adaptation to their profession [38]. Most problems are related to a wide range of potential sources of clinical learning environment and events that can lead to stress. While midwifery students usually have a good knowledge, they lack sufficient clinical skills, and they are unsuccessful in applying their theoretical knowledge in a stressful environment. This problem can be resolved with a lot of support and using a simulated environment [39, 40]. Mutual interaction, common understanding, and shared objectives are essential to students’ positive experiences in a learning environment [35]. Considering the nature of midwifery, there is a need for more focusing on orientation of students and instructors about clinical stressors [37].

It can be said although midwives say midwifery is inherently stressful, contextual and other environmental factors cause stress in midwives [9]. The students’ relationship with instructors and different aspects of the learning environment can be the most stress-inducing [41]. Iranian midwifery students have a little chance of acquiring independent experiences in clinical learning. This leads to unpleasant fears and experiences, and stress [8]. So, the support through instructors and clinical staff is essential for positive learning experiences [12].

When students are been publicly criticized by their clinical instructor, this situation causes fear and increased their stress. This issue sometimes occurs for the Iranian students, and the clinical instructors do not pay enough attention to this issue [8, 13]. An effective clinical instructor should be able to create an appropriate communication and a supportive emotional climate to produce a favorable environment for learning [5, 27]. Education process should not making distress for the learner, especially humiliating and disrespecting a student [1]. Experienced instructors are aware of how to communicate effectively with students and they do the punishment and advisement in the right time and right place. If the clinical instructor notifies to the students these matters publicly, it can damage students’ self-confidence and personality [4, 27]. The gap between theory and practice, and the midwives’ negative view to clinical educators, may also be one of the causes of ineffective clinical education. This issue can also make educational stress for students. Lukasse et al. showed that many midwives have a negative view to midwifery instructors who have not been in clinical practice for many years or who are not up-to-date in clinical practice. Old and outdated teaching methods lead to a gap between theory and practice [42].

Making a stressful relationship between the instructors and the Iranian students may be due to large number of students in clinical setting, or the use of inexperienced instructors who are not familiar with communication skills [4, 6]. It seems that the clinical environment do not provide enough support for the students studied.

In present study, the students who were interested in midwifery perceived the lower level of stressors in “clinical practices” and “interpersonal communication” dimensions. Mansouri et al. [43] and Alizadeh and Sigarchian [44] showed that the students’ interest in their field positively impacts on their academic success. Midwifery students face a high level of stress in their clinical training that can affect their mental health and academic achievement [44]. Since in most cases, enrolment in the bachelor of midwifery program is the student’s first exposure to university and hospital systems, the students have no enough knowledge about the difficulties of midwifery profession. This situation causes increasing to new concerns [45]. Therefore, practices which promote students’ interest could lead to improved academic outcomes [43]. Awareness of these issues and the factors influencing students’ interest are important for curriculum planners as well as nursing and midwifery educators [43].

### Limitations

As with all research, our study has limitations. The small sample, conducting the study at one university, and cross sectional survey design are the limitations of this study which can affect the generalizablity. However, to our knowledge, this is the first study to investigate perceived stress, and sources of stress in four different dimensions of clinical learning of midwifery students, and simultaneously has examined the relationship between stress with characteristics of the students, instructors, and clinical environment.

## Conclusions

The present study showed that midwifery students were affected by high stress, especially in “unpleasant emotions” and “humiliating experiences” dimensions. Meanwhile, the stressors associated with the instructors have caused more students’ stress. Because of the nature of midwifery profession and the specific features of midwifery education, the clinical learning of midwifery students is associated with high stress, and culture of clinical training environment seems non-supportive. This highlights the need to clearly describe the current clinical education program; preparation and support given to clinical instructors. Although education in midwifery is associated with the experience of invasive, risky and stressful clinical work, it seems that the unpleasant feelings and humiliating experiences due to ineffective student-instructor communication seem to have caused more stress in Iranian students. This can also highlights the need to preparation and support given to clinical instructors in similar cultures in other countries.

Based on our findings, the enrollment of enthusiastic students can contribute to less experience of stress in “clinical practices” and “interpersonal communication”. Since in most cases, enrolment in the bachelor of midwifery program is the student’s first exposure to university and hospital systems, the students have no enough knowledge about the difficulties of midwifery profession. This situation causes increasing to new concerns. These issues may leads to declines in students’ interest in the field of study and increasing their stress level during clinical learning. It seems that students who are interested in their field of study may be more focused on training clinical activities, expect to earn more academic achievement, and may be having better mental health and therefore would be experienced less stress.

Instructors need to increase students’ awareness of these stressors and use supportive strategies through open communication in the clinical setting. It is recommended that school managers will assess the students’ stress during clinical learning, and employ experienced instructors, and improve communication skills of the clinical instructors. Future studies are recommended to evaluate concurrently the views of students, instructors, midwives and school managers using larger sample.

## Data Availability

The datasets generated during and/or analyzed during the current study are available from the corresponding author on reasonable request.

## Abbreviations

ANOVA: Analysis of Variances
PQSS: Persian Questionnaire of Stressful Sources
PSS: Perceived Stress Scale
